# Polyphyllin VII attenuated RANKL-induced osteoclast differentiation via inhibiting of TRAF6/c-Src/PI3K pathway and ROS production

**DOI:** 10.1186/s12891-020-3077-z

**Published:** 2020-02-19

**Authors:** Long Zhou, Hanyi Song, Yiqi Zhang, Zhaozhou Ren, Minghe Li, Qin Fu

**Affiliations:** 1 Department of Orthopedics, Shengjing Hospital of China Medical University, No. 36 Sanhao Street, Heping District, Shenyang, 110004 Liaoning Province China; 2 Department of Gastroenterology, Shengjing Hospital of China Medical University, Shenyang, China; 3 Department of Clinical Oncology, Shengjing Hospital of China Medical University, Shenyang, China

**Keywords:** Polyphyllin VII, Bone marrow macrophages, Differentiation, osteoclast, ROS

## Abstract

**Background:**

Osteoporosis is a worldwide severe bone disease. This study aimed to evaluate the effect of polyphyllin VII on the genesis of osteoclasts from bone marrow macrophages (BMMs) and its potentiality as a therapeutic drug for osteoporosis.

**Methods:**

BMMs were induced to differentiate into osteoclasts by RANKL and M-CSF. The cells were then treated with various concentrations of polyphyllin VII. Intracellular reactive oxygen species (ROS) measurement assay, resorption pit formation assay, tartrate-resistant acid phosphatase (TRAP) staining and TRAP activity assessment, cell viability assay, active GTPase pull-down assay, immunofluorescent staining, immunoblotting, and RT-PCR were performed.

**Results:**

RANKL + M-CSF significantly increased TRAP activity, number of osteoclasts, number and area of lacunae, intracellular content of ROS, protein levels of Nox1, TRAF6, c-Src and p-PI3K, as well as the content of activated GTP-Rac1, which were significantly blocked by polyphyllin VII in a concentration-dependent manner.

**Conclusion:**

These findings suggested that polyphyllin VII inhibited differentiation of BMMs into osteoclasts through suppressing ROS synthesis, which was modulated by TRAF6–cSrc–PI3k signal transduction pathway including GTP-Rac1 and Nox1. Polyphyllin VII could be a therapeutic drug for osteoporosis.

## Background

Osteoporosis is characterized by reduced bone mass and disruption of bone architecture, resulting in increased risk of fragility fractures which represent the main clinical consequence of the disease. Fragility fractures are associated with substantial pain and suffering, disability and even death for affected patients and substantial costs to society [1]. It is estimated that approximately 40% white women with ages over 50 years old [2] and 13% white men with ages over 50 years [3] will suffer bone fractures due to osteoporosis. The annual economic burden for fractures derived from osteoporosis was 130–200 billion US dollars in the United States in 2005, and will increase to 250 billion US dollars in 2025 since the incidence of osteoporosis related fractures increases quickly [4]. With the increase of senior population in the society, osteoporosis and its related fractures have become a world widely concerned health problem.

The main clinically applied medications for osteoporosis are bisphosphonates, estrogen, selective estrogen receptor modulators (Raloxifene), teriparatide, Denosumab, and calcitonin. However, every medication has limitation. For example, bisphosphonates, the first line medication, have the side effects of causing necrosis of jaws, severe musculoskeletal pain, esophagus cancer, and kidney failure [5]. The newly approved RANKL inhibitor, Denosumab (Prolia), inhibits the rebuild of bone, and hence has the risk of increase in necrosis of jaws, causing atypical fractures and delaying bone repair [6]. Moreover, these medications are less effective for age, corticosteroids and other disease related osteoporosis. Therefore, development of safe and effective medications to treat osteoporosis is in high demand.

Reactive oxygen species (ROS) are small molecules containing oxygen, resulting from the oxidative metabolism processes [7]. Age and inflammation increase the production of ROS and lead to oxidative stress resulting from the imbalance between anti-oxidants and oxidants in the cells and tissues [8]. Menopause and decline in the concentration of estrogen are thought to be key factors of osteoporosis. However, it is currently found that age is the key factor causing loss of bone mass and decrease in bone strength, while decline in estrogen is just a consequence of age [9]. ROS has been found to play important roles in causing age-related osteoporosis through promoting genesis of osteoclasts and bone resorption [10].

Extracts from natural plants are attracting in that they have fewer side effects. In addition, many natural extracts from plants, including Fisetin [11], total saponin from Anemone flaccida Fr. Schmidt [12], Genistein [13], Scoparone [14], and Icaritin [15], have not only anti-oxidant effect, but also effect of anti-osteoclast genesis. These natural extracts have been reported to modulate varying signal transduction pathways including NF-κB, MAPKs, and TRAF6/c-Src/PI3K signal pathways, to reduce production of ROS, and to suppress genesis of osteoclasts [12–15]. Paris polyphylla, also known as Paris polyphylla Smith, is a lily plant. It has been found that Paris polyphylla has many therapeutic effects, such as hemostasis, anti-tumor [16], sedation, immunomodulation, and anti-parasitic effect [17]. Paris Phyllin VII, which is a natural saponin isolated from Paris polyphylla, has been reported to have anti-tumor effect [18–20] and anti-oxidation effect. It could suppress ovary cancer through removal of ROS when combined with silicon oxide nano [21]. However, no studies have been reported to investigate the roles of Paris polyphylla in resisting osteoporosis. Therefore, in this study, using in vitro cell culture, the inhibitory effect of Paris Phyllin VII on osteoclast genesis and the underlying mechanisms were explored.

## Materials and methods

### Isolation of bone marrow macrophages (BMMs) from mice

Ethical approval was approved by the Ethics Committee of Shengjing Hospital of China Medical University, Shenyang, China. ICR mice with ages of 4–6 weeks (Beijing Huakangkang Biotechnology Co., Ltd., Beijing, China) were sacrificed by skull relocation. Two hind leg bones were dissected out on a sterile station. Three ml of α-MEM complete media containing 10% serum was added to a 6 cm dish, and the cells in the bone marrow cavity were blew out into the dish three times using a 1 ml syringe. The cells were cultured in an incubator at 37 °C for 20 h (overnight). The cells in the super cell suspension was harvested, counted and used as primary BMMS.

### Cell viability assay

BMMs (3 × 10^3^) in 100 μl of DMEM containing 10% bovine serum were plated into each well of a 96-well plate and incubated in an incubator containing 5% CO_2_ at 37 °C overnight. Next day, polyphyllin VII (Solarbio, Beijing, China), which was dissolved with DMSO at stock solution and further diluted with medium to final concentration of 0 μM, 1 μM, 10 μM, 30 μM, or 50 μM, was added into the culture. The cells were further incubated for 9 days, and the cytotoxic effect of polyphyllin VII on the cells was tested using CCK-8 test kit (Dojindo, Japan) following the manufacture’s instruction.

### Tartrate-resistant acid phosphatase (TRAP) staining and TRAP activity test

BMMs were plated into a 96-well plate at a density of 1 × 10^4^ cells/well and grouped as negative control group without any treatment, or experimental groups that were treated with 20 ng/ml of RANKL, 20 ng/ml of M-CSF and polyphyllin VII at the concentrations of 0, 1, 10, 30 and 50 μM. The media containing the corresponding induction reagents were changed once on the third day after seeding and once every other day during the following culture period. When osteoclasts were formed during 7–9 days in culture, the cells were fixed and stained with TRAP using commercial kit (Sigma-Aldrich, Cat. no. 387) following the manufacture’s instruction. The TRAP positive cells with multi-pseudopodia and multi-nuclei (> 3 nuclei) were counted. The activity of TRAP was determined using tartrate-resistant acid phosphatase assay kit (P0332, Beyotime Biotechnology, Shanghai, China) following the manufacture’s instruction.

### Resorption pit formation assay

Resorption pit formation assay was performed on Corning 96-well plate (Corning, 3989, USA). Briefly, BMMs were seeded into a 96-well hydroxyapatite plate at a density of 1 × 10^3^ cells/well, grouped as negative control group without any treatment, experimental groups that were treated with 20 ng/ml of RANKL, 20 ng/ml of M-CSF and polyphyllin VII at the concentrations of 0, 1, 10, 30 and 50 μM for 9 days. The media containing corresponding induction reagents were changed once on the third day after seeding and once every other day during the following culture period. When osteoclasts were induced (9 days induction), the media were discarded and replaced with 100 μL of 10% bleach. After standing at room temperature for 5 min, bleach was discarded, and cells were washed with ddH_2_O twice for 5 min each. They were air-dried at room temperature for 3–5 h. The resorption pits formed on the hydroxyapatite plate due to erosion by osteoclasts were observed and photographed under an invert microscope. Average areas of resorption lacunae per well was measured and expressed as pixel dimensions.

### Assessment of intracellular ROS

BMMs were seeded into a 96-well plate at a density of 1 × 10^4^ cells/well. The cells were grouped as negative control group without any treatment and experimental groups that were treated with 20 ng/ml of RANKL, 20 ng/ml of M-CSF and polyphyllin VII at the concentrations of 1, 10, and 30 μM. After 48 h induction, cells were washed with PBS twice, and 2′-7′-Dichlorodihydrofluorescein diacetate (DCFH-DA) was added in a final concentration of 50 μM. After incubation at dark at 37 °C for 30 min, cells were washed with PBS three times and treated with trypsin solution containing no phenol red. Cells were suspended in 500 μL of PBS, centrifuged at 1000 rpm, and re-suspended in 500 μL of PBS. The fluorescence from DCFH-DA bound to ROS was measured using flow cytometry.

### Quantitative real-time RT-PCR (qRT-PCR)

Real time qRT-PCR was performed as previously reported procedure to quantify the mRNA of serum band 5 tartrate-resistant acid phosphatase (TRACP5), cathepsin K (CtsK), and nuclear factor of activated T-cell cytoplasmic 1 (NFATc1) [22]. Briefly, total RNA was extracted using RNAiso Plus (Takara Biotechnology Co., Ltd., Dalian, China). Reverse transcription was performed using Prime-Script RT reagent kit with gDNA eraser (Takara Biotechnology Co. Ltd., Dalian, China). Quantitative real time PCR was conducted using ABI 7500 (Applied Biosystem, Foster City, CA, USA) to determine mRNA expression of the target gene. Level of gene expression was expressed as relative to GAPDH and calculated using the 2^-ΔΔCt^ method.

The primers used were as followings: Tracp5, forward primer: 5′-GTGCTGCTGGGCCTACAAAT-3′, reverse primer: 5′- TTCTGGCGATCTCTTTGGCAT-3′; Ctsk, forward primer: 5′- GAAGAAGACTCACCAGAAGCAG − 3′, reverse primer: 5′- TCCAGGTTATGGGCAGAGATT-3′; Nfatc1, forward primer: 5′-CCCGTCACATTCTGGTCCAT-3′, reverse primer: 5′-CAAGTAACCGTGTAGCTGCACAA-3′, and GAPDH, forward primer, 5′-ACCCAGAAGACTGTGGATGG-3′, reverse primer: 5′-TTCAGCTCAGGGATGACCTT-3′.

### Active GTPase pull-down assay

Rac1-GTP pulled down assay was performed using Active GTPase Pull-down and Detection Kit (Thermos Scientific) following the manufacture’s instruction followed by immunoblotting for the target.

### Immunoblotting

BMMs were pretreated with various concentrations of polyphyllin VII followed by inducing to osteoclasts with RANKL and M-SCF in the presence of varying concentrations of polyphyllin VII for 9 days. Cells were then harvested with RIPA containing inhibitors of proteases and phosphatase. Protein concentration was determined using BCA method. Proteins were differentiated by 10% SDS-PAGE gels, transferred onto PDF membrane, and immunoblotting was performed using the following primary antibodies: anti-PIK3, −p-PIK3 antibodies (Cell Signaling Technology); −TRAF6, −c-Src, −Rac1, and Nox1 antibodies (Abcam); and –ß-actin antibody (Proteintech). After reacting with appropriate 2nd antibodies, the protein bands were visualized by luminescent liquid and photographed. Band density was analyzed using Image J software.

### Statistical analysis

Statistical analysis was performed using SPSS 17.0 software. One-way ANONA was used to analyze the differences among groups. If there was statistical difference between groups, the statistical significance was further determined by Tukey test, and comparison to control group was analyzed with Dunnett method. All data were expressed as mean ± SD. *P* < 0.05 was considered as statistically significant.

## Results

### Effects of polyphyllin VII on BMM cell survival

**Fig. 1:**
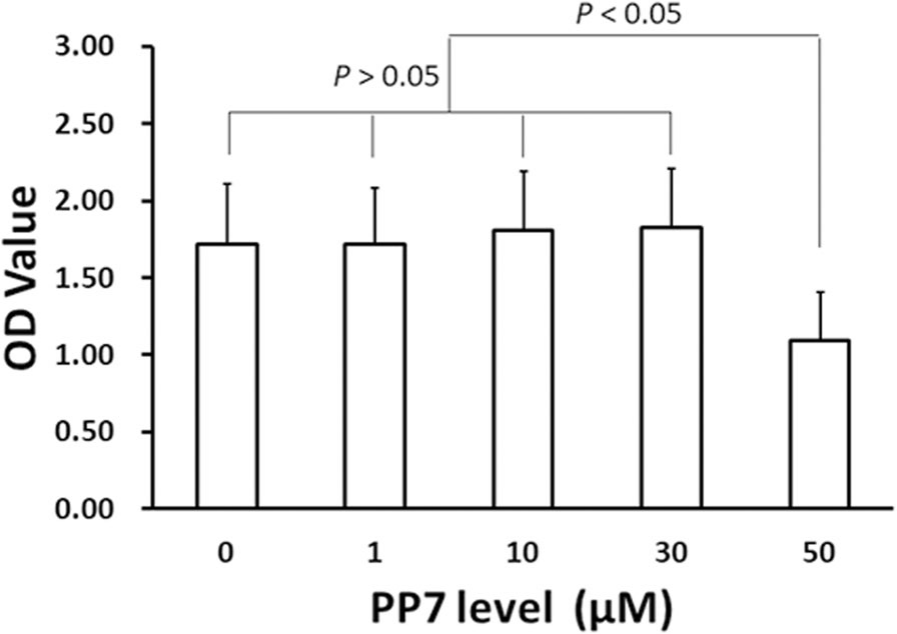
Cytotoxicity of polyphyllin VII on the BMMs cells. BMMs cells were cultured in the media containing various concentrations of polyphyllin VII for 9 days. The effects of polyphyllin VII on BMMs cells were measured using CCK-8 reagent kit as described in the methods. Vertical axis: OD value; horizontal axis: polyphyllin VII (PP7) concentration (μM)

Polyphyllin VII at the concentrations of 1, 10 or 30 μM did not cause significant cell death of BMMs (Fig. 1). At 50 μM, however, polyphyllin VII induced significant (*P* < 0.05) cell death in the BMMs (Fig. 1). Therefore, polyphyllin VII was used at a concentration of 30 μM or less for the rest of experiments in this study.

### Inhibitory effect of polyphyllin VII on the genesis of osteoclasts

**Fig. 2:**
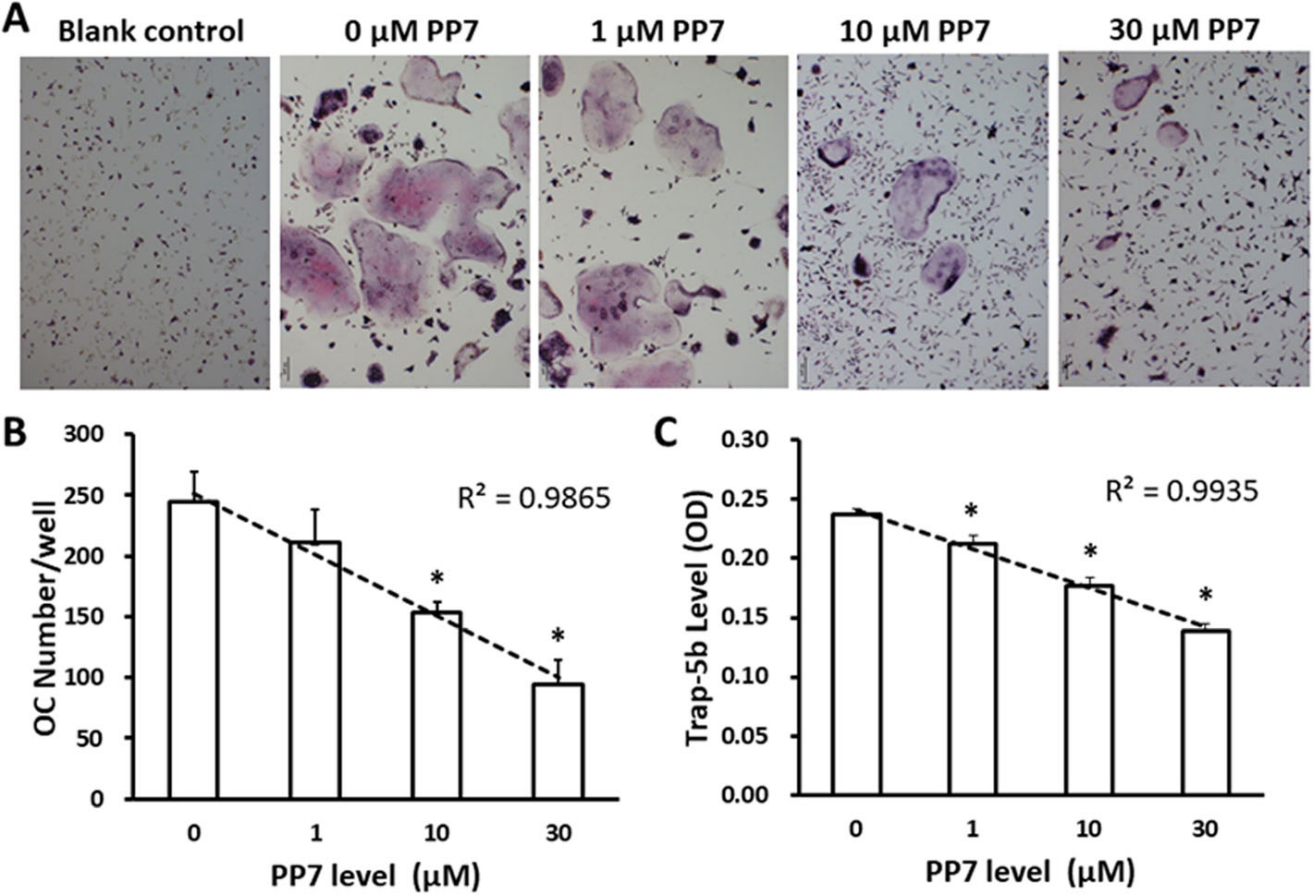
Inhibitory effect of polyphyllin VII (PP7) on the osteoclast differentiation from BMMs. BMMs were induced to differentiate into osteoclasts with or without RANKL and M-CSF in the presence of various concentrations of polyphyllin VII (PP7). The osteoclasts were visualized by TRAP staining. **Panel a** BMMs cells stained with TRAP. Cells with multiple red nuclei indicated osteoclasts. Magnification: × 100. **Panel b** Number of TRAP positive cells in each well. Vertical axis: osteoclast cell number; horizontal axis: polyphyllin VII (PP7) concentration (μM). **Panel c** Relative activity of TRAP in polyphyllin VII-treated cells compared to the control cell. Vertical axis: TRAP-5B level; horizontal axis: polyphyllin VII (PP7) concentration (μM). * *P* < 0.05, compared to the cells treated with 0 μM of polyphyllin VII (PP7); the dotted line represents a regression line

BMMs were treated with RANKL and M-CSF for 2 h followed by further treatment in the presence of various concentrations of polyphyllin VII for additional 9 days. TRAP expression and TRAP activity were then assessed in the differentiated osteoclasts. It was found that number of the osteoclasts differentiated from BMMs significantly (*P* < 0.05) decreased in the presence of 10 μM or 30 μM of polyphyllin VII (Fig. 2a, b), and that TRAP activity was also significantly suppressed by polyphyllin VII in a concentration-dependent manner (1,10 and 30 μM, Fig. 2c).

### Inhibitory effect of polyphyllin VII on resorption capacity of osteoclasts

**Fig. 3:**
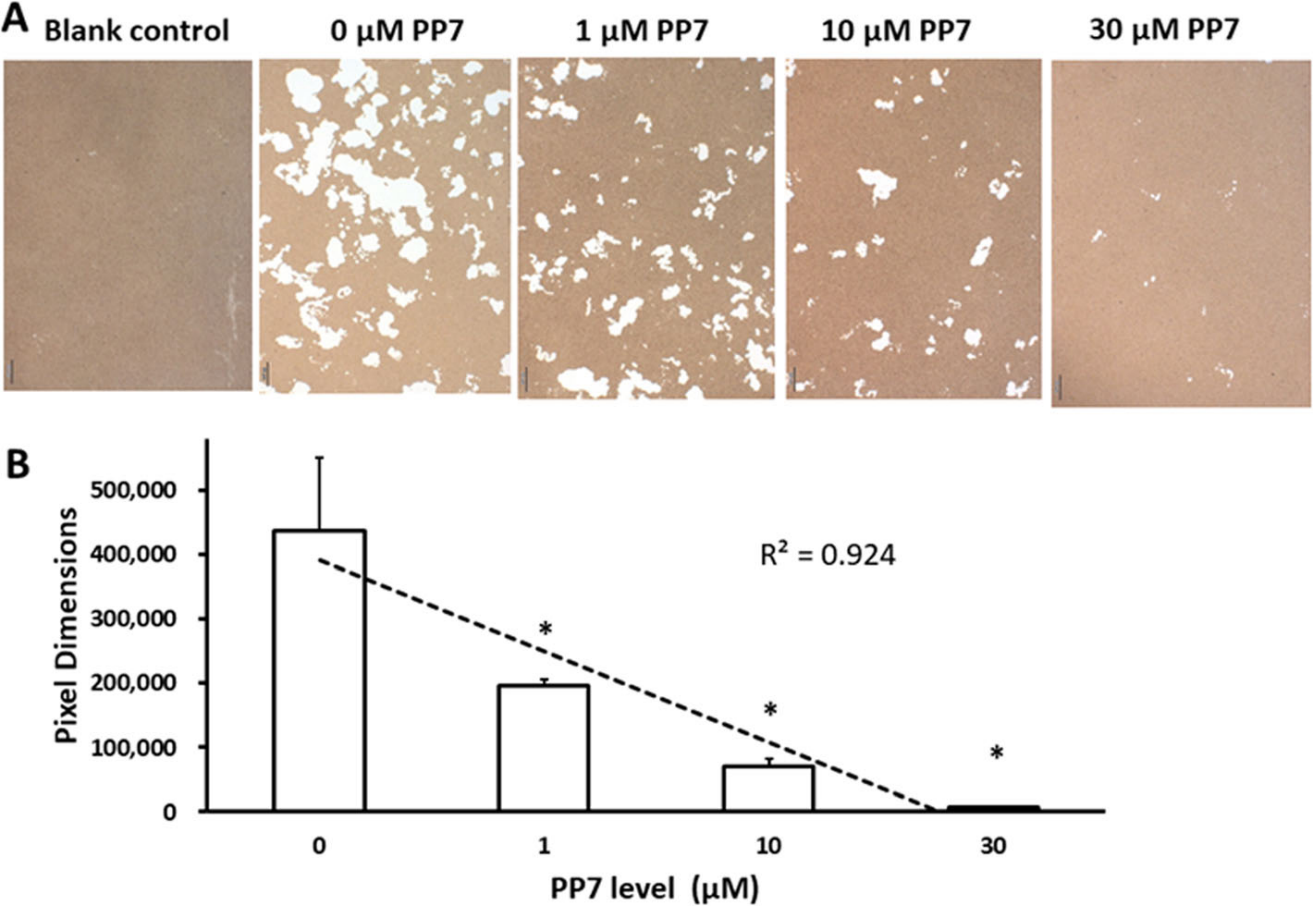
Inhibitory effect of polyphyllin VII (PP7) on the resorption capability of osteoclasts. **Panel a** Image of resorption lacunae under light microscope. The resorption lacunae assay in the presence of various concentrations of polyphyllin VII (PP7) was performed as described in the methods. **Panel b** Average areas of resorption lacunae per well in the presence of various concentrations of polyphyllin VII (PP7). Vertical axis: pixel dimensions; horizontal axis: polyphyllin VII (PP7) concentrations (μM). * *P* < 0.05 as compared with the group treated with 0 μM of polyphyllin VII (PP7); the dotted line represents a regression line

Resorption lacunae are result of bone resorption and a marker of resorption capacity. RANKL increased the number and area of lacunae (Fig. 3a), which was significantly suppressed by polyphyllin VII (*P* < 0.05) in a concentration-dependent manner (Fig. 3a, b).

### Inhibitory effect of polyphyllin VII on the mRNA expression of TRACP5, Ctsk and NFATc1

**Fig. 4:**
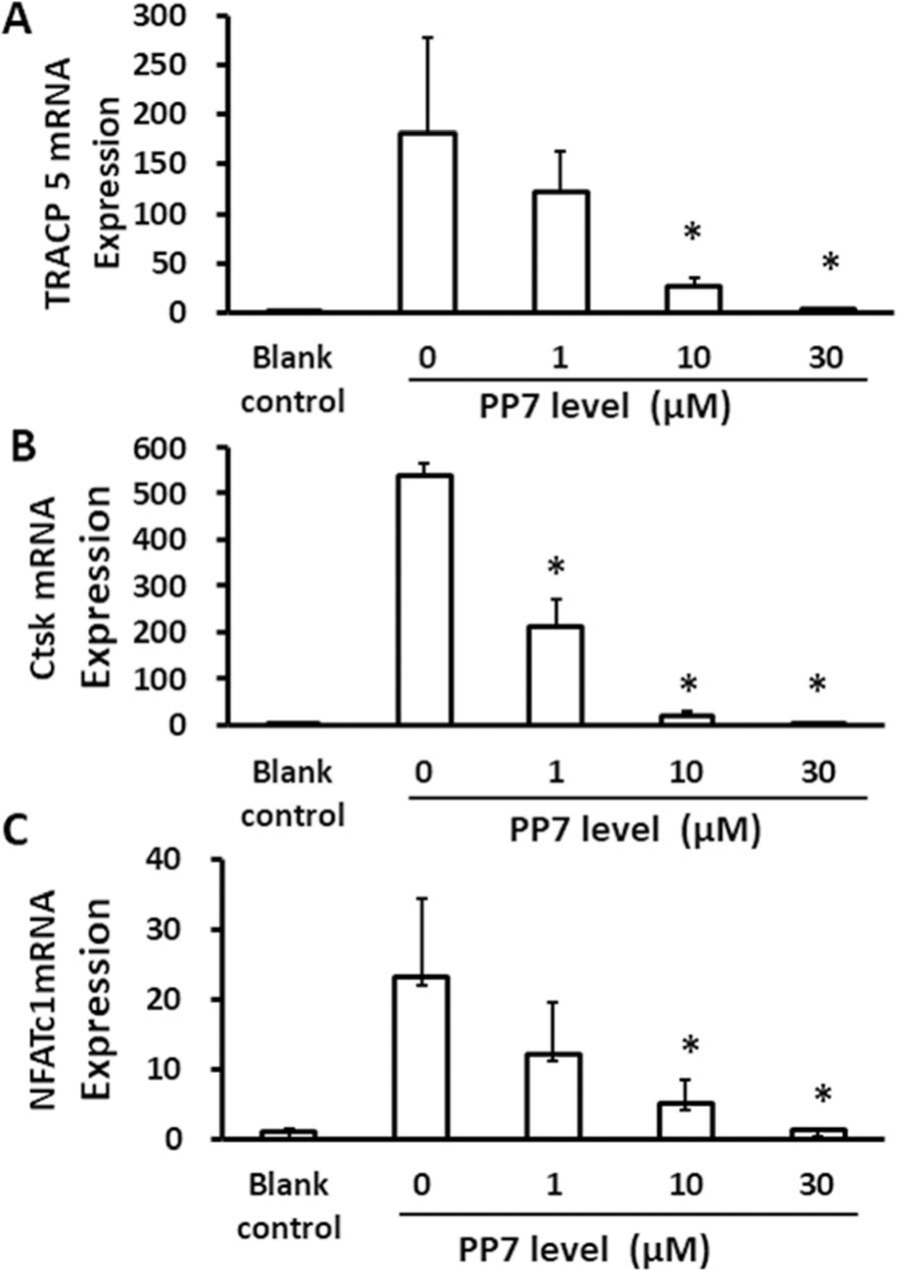
Effect of polyphyllin VII (PP7) on gene expression of TRACP5, Ctsk, and NFATc1 in the differentiated osteoclasts. After pretreatment with various concentrations of polyphyllin VII, BMMs were induced to differentiate into osteoclasts in the presence or absence of RANKL and M-CSF. After 24 h treatment, mRNA levels of TRACP5, Ctsk, and NFATc1 were quantified using RT-PCR as described in the methods. **Panel a** TRACP5 mRNA expression. Vertical axis: TRACP5 mRNA expression; horizontal axis: polyphyllin VII (PP7) concentrations (μM). **Panel b** Ctsk mRNA expression. Vertical axis: Ctsk mRNA expression; horizontal axis: polyphyllin VII (PP7) concentrations (μM). **Panel c** NFATc1 mRNA expression. Vertical axis: NFATc1 mRNA expression; horizontal axis: polyphyllin VII (PP7) concentrations (μM). * *P* < 0.05 as compared to the cells treated with 0 μM of polyphyllin VII. Blank control: BMM cells without RANKL or PP7 treatment

As shown in Fig. 4, gene expression of TRACP5 (Fig. 4a), Ctsk (Fig. 4b) and NFATc1 (Fig. 4c) was significantly increased in the BMM cells exposed to RANKL compared to the cells without any treatment (*P* < 0.05). Polyphyllin VII significantly (*P* < 0.05) inhibited mRNA expression of TRACP5 at the concentrations of 10 and 30 μM (Fig. 4a), of Ctsk at the concentrations of 1, 10 and 30 μM (Fig. 4b), and of NFATc1 at the concentrations of 10 and 30 μM (Fig. 4c).

### Inhibitory effect of polyphyllin VII on production of ROS during differentiation of BMMs to osteoclasts

**Fig. 5:**
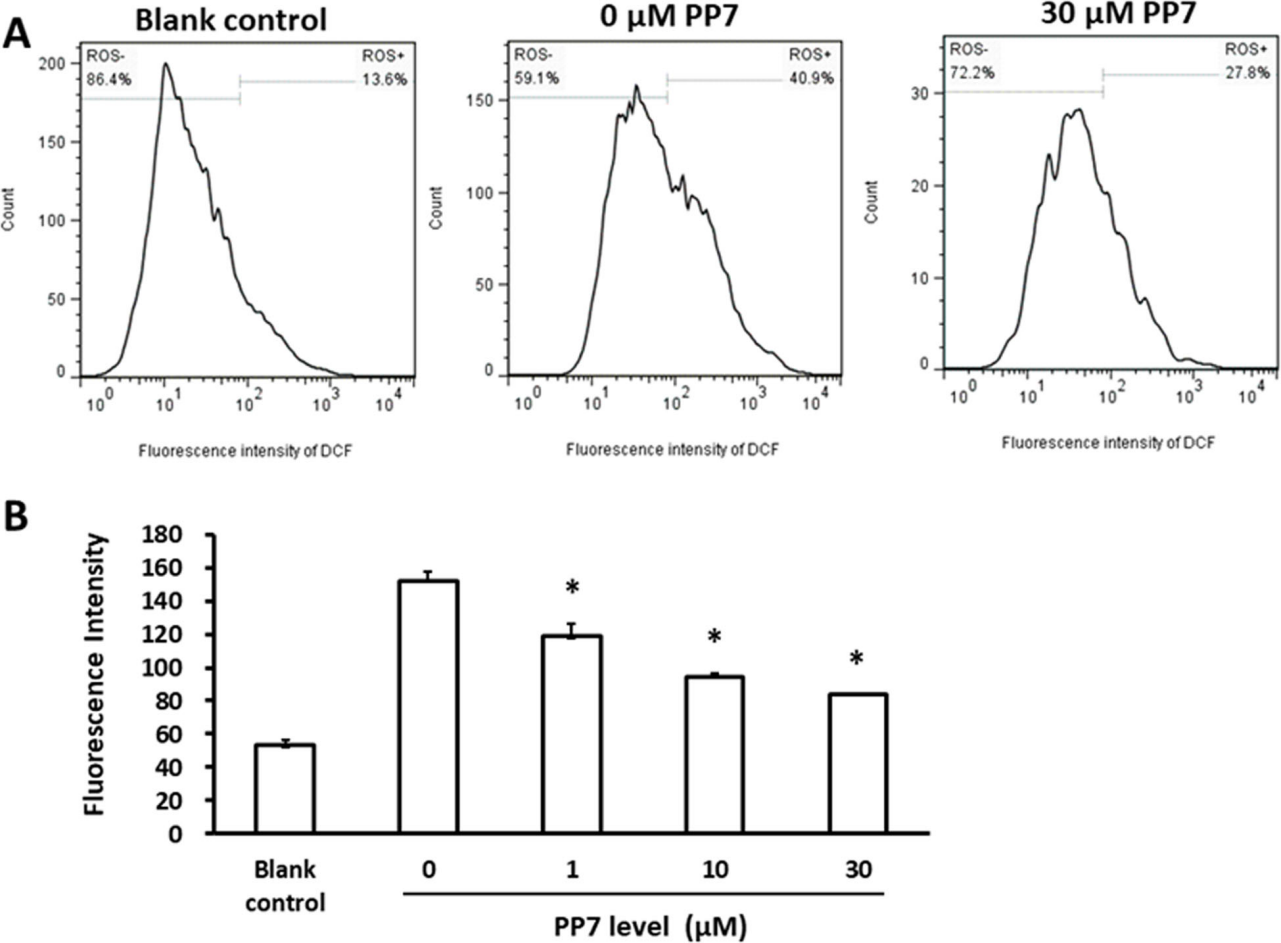
Inhibitory effect of polyphyllin VII (PP7) on ROS generation in the differentiated osteoclasts. BMMs cells were pretreated with various concentrations of PP7 for 2 h. Cells were then induced with or without RANKL plus M-CSF to differentiate into osteoclasts. Intracellular ROS was measured using flow cytometry. **Panel a** Representative plots of ROS measured with flow cytometry. **Panel b** Comparison of the fluorescence intensity of DCF among cells treated with various concentration of polyphyllin VII (PP7). Vertical axis: fluorescence density; horizontal axis: polyphyllin VII (PP7) concentration (μM). * *P* < 0.05, as compared with the cells treated with 0 μM of polyphyllin VII

It has been reported that ROS plays an important role in the differentiation of osteoclasts [10]. Since polyphyllin VII is a natural antioxidant, it is perceptible that polyphyllin VII may inhibit the differentiation of osteoclasts via its effect of anti-oxidation. Therefore, effect of polyphyllin VII on ROS production was assessed in this study. It was found that intracellular content of ROS in the BMMs was significantly increased in response to RANKL and M-CSF stimulation (Fig. 5a, *P* < 0.05), which was significantly suppressed by polyphyllin VII in a concentration-dependent manner (*P* < 0.05, Fig. 5b).

### Inhibitory effect of polyphyllin VII on TRAF6/c-Src/PI3K /Nox1 pathway

**Fig. 6:**
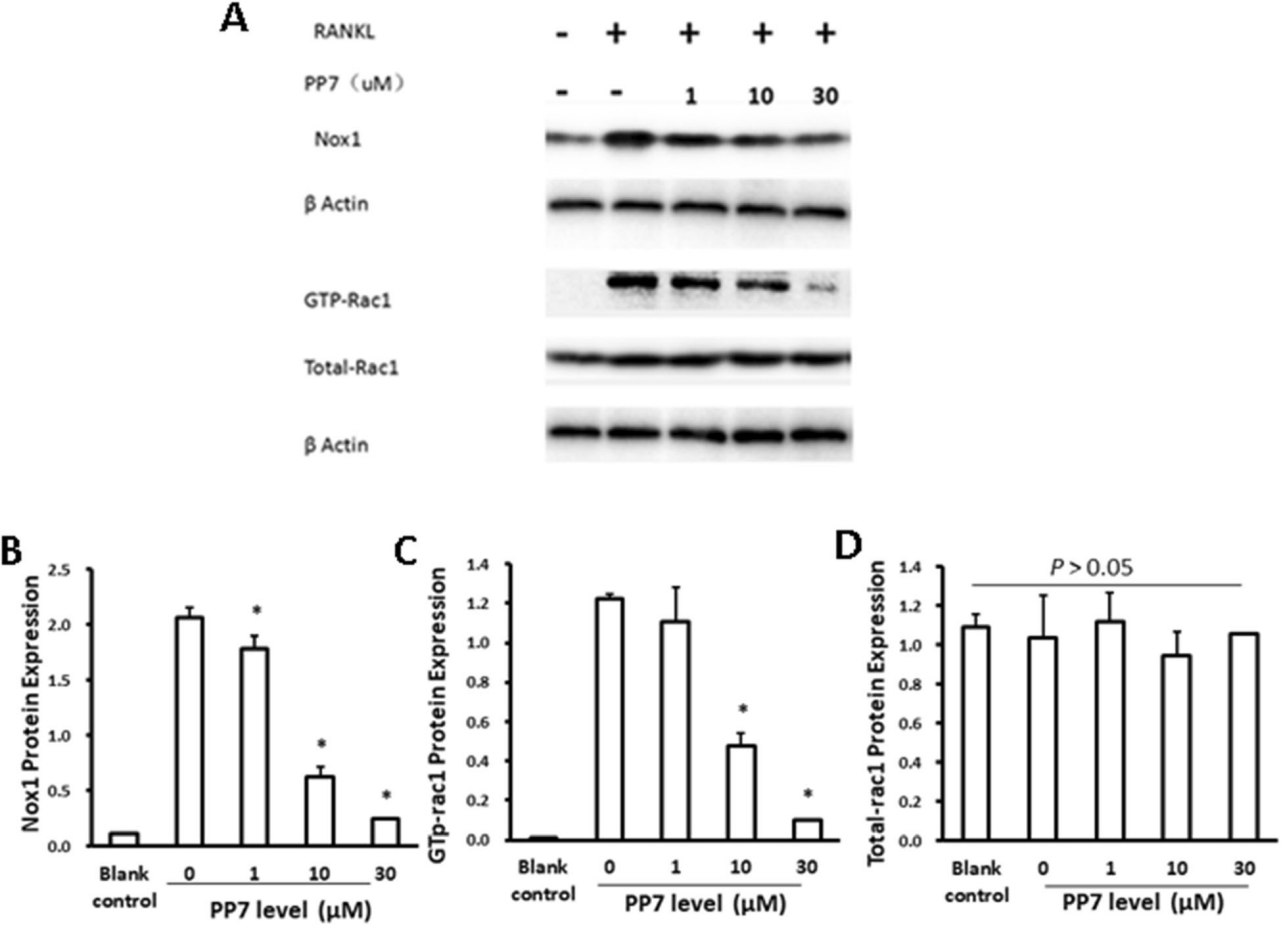
Protein levels of Nox1, GTP-Rac1 and Total-Rac1. BMMs cells were pretreated with various concentrations of polyphyllin VII (PP7) for 2 h. The cells were then induced to differentiate into osteoclasts with or without RANKL and M-CSF. After 48 h, protein levels of Nox1, GTP-Rac1, and Total-Rac1 were assessed by immunoblotting as described in the methods. **Panel a** Representative image of immunoblotting assay of Nox1, GTP-Rac1 and Total-Rac1 protein. **Panel b, c and d:** Effect of polyphyllin VII (PP7) on the expression of Nox1 protein (**b**), GTP-Rac1 protein (**c**), and Total-Rac1 protein (**d**). Vertical axes: protein level; horizontal axes: polyphyllin VII (PP7) concentrations (μM). * *P* < 0.05 as compared to the cells treated with 0 μM of polyphyllin VII

It has been reported that cellular ROS production was highly related with Nox1 [23, 24], and that Nox1 could only be activated by combination with GTP-Rac1 in order to stimulate the generation of ROS [23]. Therefore, effect of polyphyllin VII on Nox1 and GTP-Rac1 activation was assessed in the current study. It was found that RANKL increased protein level of Nox1, while polyphyllin VII significantly (*P* < 0.05) inhibited RANKL-mediated increase of Nox1 in a concentration-dependent manner (Fig. 6a, b). RANKL increased the content of activated GTP-Rac1 given the total content of Rac1 was constant (Fig. 6a, d), while polyphyllin VII significantly (*P* < 0.05) inhibited the RANKL-mediated increase in the content of GTP-Rac1 in a concentration-dependent manner (Fig. 6a, c).

**Fig. 7:**
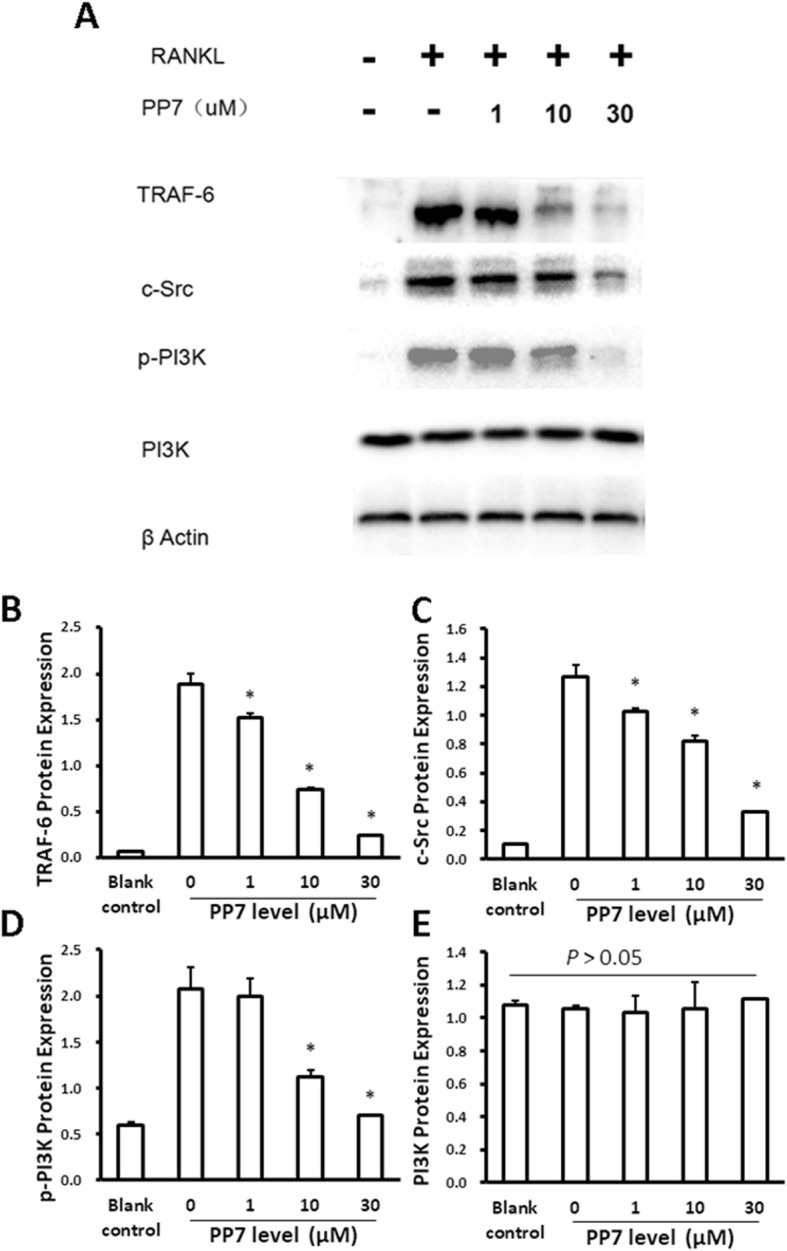
Effect of polyphyllin VII on components of the TRAF6/c-Src/PI3K signal transduction pathway. BMMs cells were pretreated with various concentrations of polyphyllin VII (PP7). The cells were the induced to differentiate into osteoclasts with or without RANKL and M-CSF. After 48 h, protein levels of the molecules in RAF6/c-Src/PI3K signaling pathway were assessed with immunoblotting. **Panel a** Representative image of immunoblotting of TRAF6, c-Src, p-PI3k and PI3k protein. **Panel b, c, d and e** protein expression level of TRAF-6 (**b**), c-Src (**c**), p-PI3K (**d**), and PI3K (**e**). Vertical axes: protein expression level; horizontal axes: polyphyllin VII (PP7) concentrations (μM). * *P* < 0.05 as compared to the cells treated with 0 μM of polyphyllin VII

It has also been reported that activation of Nox1 was regulated by the upstream TRAF6/c-Src/PI3K pathway [25–27], and thus, effect of polyphyllin VII on the TRAF6/c-Src/PI3K pathway was further explored. RANKL increased the protein levels of TRAF6 and c-Src, as well as the level of phosphorylated PI3K (p-PI3K, Fig. 7a-d), while it did not alter the level of PI3K (Fig. 7e). Polyphyllin VII significantly inhibited the RANKL-induced increase of TRAF6 and c-Src protein expression, as well as the level of p-PI3K (Fig. 7a-d) in a concentration-dependent manner without affecting the level of PI3K (Fig. 7e).

## Discussion

It has been reported that mouse BMMs could be induced to differentiate into osteoclasts through RANK activation [28]. In this study, role of polyphyllin VII on the differentiation of BMMs into osteoclasts in response to RANKL plus M-CSF was investigated. RANKL increased TRAP activity, number of osteoclasts, number and area of lacunae, content of ROS, protein levels of Nox1, TRAF6, c-Src and p-PI3K, as well as the content of activated GTP-Rac1, which were significantly blocked by polyphyllin VII in a concentration-dependent manner. These results suggested that polyphyllin VII attenuates RANKL-induced osteoclastic genesis via the inhibition of TRAF6/c-Src/PI3K pathway and production of ROS.

### Polyphyllin VII inhibited RANKL-induced genesis of osteoclasts from BMMs

Polyphyllin VII did not affect cell survival of BMMs at the concentrations of 1 μM, 10 μM or 30 μM while Polyphyllin VII caused cytotoxicity at 50 μM. This observation indicated that polyphyllin VII at a concentration of 30 μM or lower was safe for the cells, and thus, polyphyllin VII at a concentration of 30 μM or lower was used in this study. After BMMs were induced with RANKL and M-CSF, the number of TRAP positive cells and the activity of TRAP were increased, suggesting that BMMs were induced to differentiate into osteoclasts. Resorption lacunae are result of bone resorption and a marker of resorption capacity. Increase of the number and area of lacunae in response to RANKL treatment indicated that the RANKL-induced TRAP positive cells were functional osteoclasts.

It has been reported that TRACP5, Ctsk, NFATc1 were the markers of differentiation of BMMs into osteoclasts [22]. Consistently, in the current study, we further demonstrated that mRNA levels of TRACP5, Ctsk and NFATc1 were significantly up-regulated in response to RANKL and M-CSF exposure, further demonstrating that BMMs were successfully induced to differentiate into osteoclasts.

In the presence of Polyphyllin VII, the number of TRAP positive cells, activity of TRAP, number and area of lacunae were reduced and gene expressions of TRACP5, Ctsk and NFATc1 were down-regulated. These results implicated that polyphyllin VII inhibited the differentiation of BMMs to osteoclasts.

### Polyphyllin VII modulated ROS production during differentiation of BMMs to osteoclasts

It has been reported that ROS plays an important role in the differentiation of BMMs to osteoclasts [10, 23, 29]. In the present study, therefore, cellular content of ROS was evaluated after BMMs were treated with RANKL and M-CSF. It has been reported that production of intracellular ROS was highly related with Nox1 [23, 24]. In this regard, Nox1 could only be activated by combination with GTP-Rac1 before it stimulated the generation of ROS [23]. Knockdown of Nox1 decreased RANKL-induced production of ROS and the number of osteoclasts generated by RANKL induction [23]. Consistently, we demonstrated that RANKL induced an increase in Nox1 protein level and the content of GTP-Rac1, suggesting that RANKL might increase the production of ROS through up-regulation of Nox1 in the current study. Moreover, consistent with previous reports that activation of Nox1 was regulated by the upstream TRAF6/c-Src/PI3K pathway [25–27], we further demonstrated that RANKL increased the expression of TRAF6, c-Src and phosphorylation of PI3K, suggesting the up-regulation of Nox1 by RANKL could be dependent on the activation of TRAF6–cSrc–PI3k signal. These findings suggested that RANKL might induce the genesis of osteoclasts from BMMs through increased production of ROS, which was modulated by Nox1 through activated TRAF6–cSrc–PI3k signal pathway.

Polyphyllin VII is a natural antioxidant and may neutralize ROS via its anti-oxidation capacity. In line of this concept, the current study demonstrated that polyphyllin VII attenuated intracellular ROS production in a concentration-dependent manner. Polyphyllin VII also significantly blocked RANKL-stimulated up-regulation of Nox1, GTP-Rac1, TRAF6, c-Src and phosphorylation of PI3K. These findings suggested that polyphyllin VII inhibited differentiation of BMMs into osteoclasts through suppressing intracellular ROS synthesis, which was mediated by the TRAF6–cSrc–PI3k signal pathway as well as GTP-Rac1 and Nox1. The mechanism of Nox1 regulation on the production of ROS, however, remains to be further investigated.

## Conclusions

**Fig. 8:**
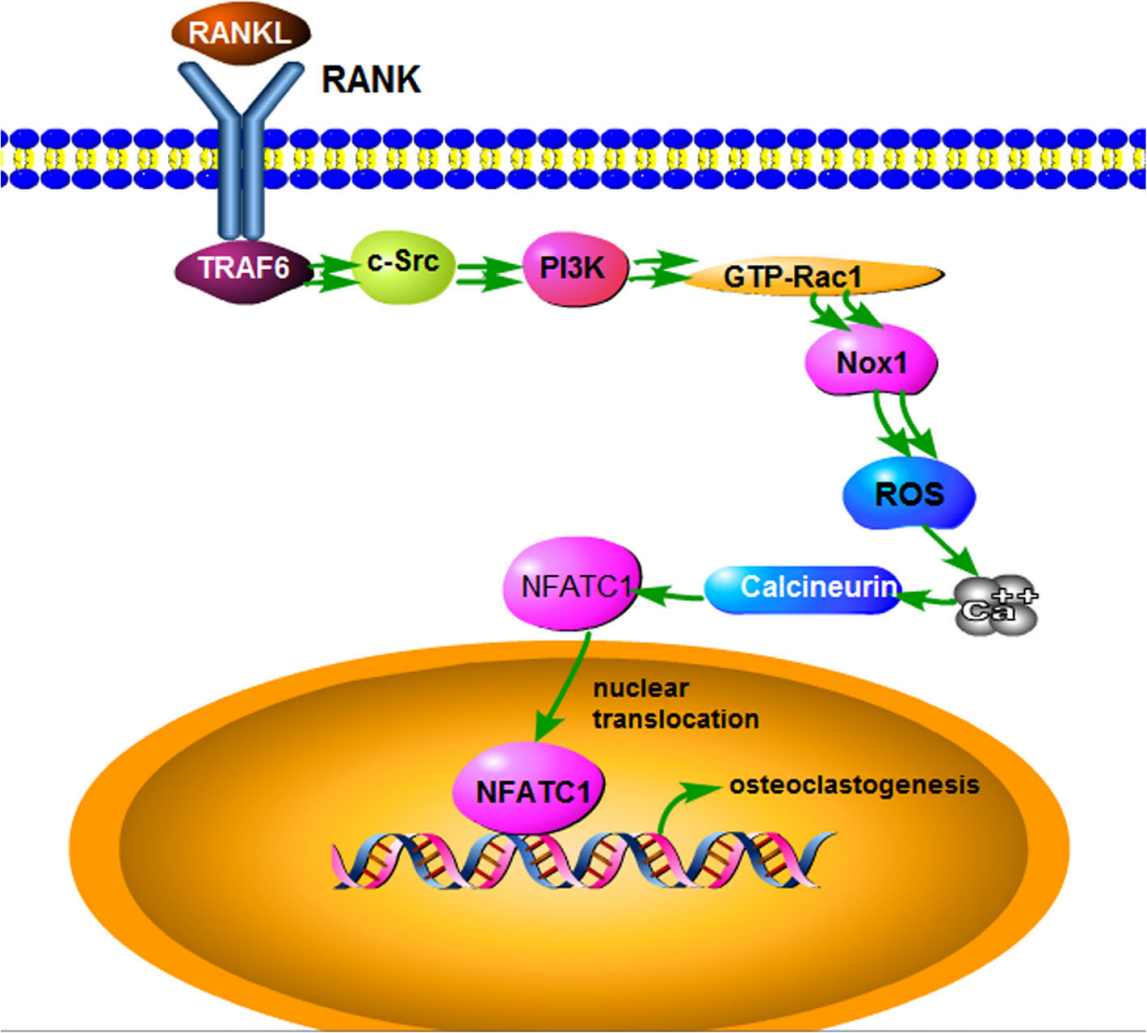
Schematic illustration of the potential mechanisms of polyphyllin VII (PP7) inhibition on BMMs differentiation into osteoclasts

Taken together, as illustrated in the Fig. 8, polyphyllin VII inhibited differentiation of BMMs into osteoclasts through suppressing synthesis of intracellular ROS, which was mediated by the signal transduction of the TRAF6–cSrc–PI3k pathway as well as activation of GTP-Rac1 and Nox1. Findings of the current study suggested that polyphyllin VII could be a therapeutic drug for osteoporosis.

## Data Availability

Part of the datasets generated and analysed during the current study are available in the ChemIndex repository, accessible through http://www.chemindex.com/index/?f=show_cas_info&terms=76296-75-8. The other data was obtained from the originally cited publications.

## Abbreviations

BMMs: Bone marrow macrophages
ROS: Reactive oxygen species
